# The Surgical Treatment of Colitis

**Published:** 1903-10-03

**Authors:** 


[1] Medical Record.

### THE SURGICAL TREATMENT OF COLITIS.

At the present time it is not usual to invoke the aid of surgery for the relief of colitis until medical treatment has had a prolonged trial and has failed. According to Dr. Gibson[1] this is a mistake, and he urges that if surgery is to succeed where medicine has failed it should be made use of at an earlier stage of the disease, before the acute condition has led to such permanent alterations in the bowel as may preclude a favourable issue. This end, he admits, can be attained only if surgical art can offer methods giving a reasonable certainty of improvement, yet devoid of the risks and possible bad after-results inherent to a serious surgical operation. In his experience it is usual in bad cases of colitis of any origin to find the whole of the large intestine involved in the inflammatory process. Topical treatment from below is bound, therefore, to be inefficient, and in order to treat the whole bowel, in those cases which do not yield to gentler measures, the aid of the surgeon may be advantageously sought. Until recently the surgical treatment of colitis consisted of making an artificial anus in order to give rest to the bowel and for the treatment of the ulcerated areas by appropriate local applications. The objections to this operation, as Dr. Gibson points out, are manifold. In the first place a simple opening into the gut gives a maximum amount of discomfort, while perfect physiological rest for the bowel below can only be obtained by a complete artificial anus necessitating for its future repair one of the severest operations practised in abdominal surgery. With a view to adopting a middle course, Dr. Gibson devised a plan of creating an artificial valvular fistula into the cæcum. This he does after the manner of Kader's gastrostomy. By this method a fistula is formed which does not leak and which will close spontaneously as soon as the daily insertion of a tube is omitted. Topical applications can be commenced on the third day after the operation. The tube can be left out at the end of a week, after which it is inserted two or three times a day in order to irrigate the large bowel and to apply local medication.

Summing up the advantages of this operation, Dr. Gibson states that the results have been such as to justify the hope of marked amelioration or cure of the symptoms. It is devoid of every disagreeable feature of an artificial anus. It calls for only a short confinement to bed. If a prolonged course of treatment is required, it may be carried out if necessary by the patient himself. A change of climate, so often beneficial, is made possible. Its performance is perfectly simple and safe. The intermuscular incision greatly reduces the risk of a subsequent hernia. The prompt closure of the fistula is certain and spontaneous.

Whether Dr. Gibson's operation will stand the test of time and experience and become an approved practice in cases of colitis remains of course to be seen, but it is certainly an ingenious and apparently rational method of treatment.

